# Determining Morphometric Differences in Domestic Fowl (*Gallus gallus domesticus* L. 1758) Tarsometatarsus Using Artificial Intelligence

**DOI:** 10.3390/ani16040530

**Published:** 2026-02-08

**Authors:** Sedat Aydoğdu, Reyhan Rabia Kök, Mustafa Zeybek, Emrullah Eken

**Affiliations:** 1 Department of Anatomy, Faculty of Veterinary Medicine, Selçuk University, 42250 Konya, Türkiye; reyhan.kok@selcuk.edu.tr (R.R.K.); eeken@selcuk.edu.tr (E.E.); 2 Guneysinir Vocational School, Selçuk University, 42250 Konya, Türkiye; mzeybek@selcuk.edu.tr

**Keywords:** domestic fowl, machine learning, morphometric measurement, tarsometatarsus

## Abstract

Artificial intelligence models, which have begun to be used in every field of science in recent years, have also started to come to the forefront in the classification of avians using bones. This study aimed to identify breeds of domestic fowl (*Gallus gallus domesticus* L. 1758) using morphometric measurements obtained from the tarsometatarsus bone and machine learning. A total of 328 tarsometatarsus specimens from two different modern domestic fowl breeds were used. A model was developed by performing 10 different morphometric measurements on each tarsometatarsus, and 3280 data points were obtained. Before model development, data cleaning and necessary assessments were carried out, and gaps were identified. In pre-processing and data partitioning, 70% of the data was used for training, and 30% was reserved for testing the developed model. To determine the differences between breeds, evaluations were performed using classical supervised learning algorithms in machine learning. Random forest (RF), support vector machine with radial kernel (SVM-RBF), and the generalized linear model (GLM, logistic regression) were used for model development, while model validation was performed using cross-validation (CV) metrics. After model validation, variable importance, feature selection, correlation analysis, dimensionality reduction, and multicollinearity were performed. The developed model, using morphological measurements obtained from the tarsometatarsus, distinguishes between breeds with high accuracy. The discriminative signal is extremely strong, allowing multiple modeling strategies (tree-based, kernel-based, and linear) to perfectly distinguish between the two breeds. Among the morphometric measurements, Ac (extension of *the trochlea metatarsi IV*) and Bmit (breadth of the middle trochlea) were found to be the strongest distinguishing features. This developed model combines morphometric data and artificial intelligence to offer an innovative method for scaling, accelerating, or improving applications in science. By expanding the model’s database with measurements obtained from the tarsometatarsus bones of different breeds, it was demonstrated that breed differences can be quickly and accurately determined using a minimal number of measurements from tarsometatarsus bones.

## Simple Summary

In domestic fowl (*Gallus gallus domesticus* L. 1758), morphometric measurements obtained from bones are extremely important parameters for breed differentiation. This differentiation is achieved using both linear measurements obtained from bones and shape analyses. Many artificial intelligence models developed in recent years have begun to be used in various fields of science. In this study, differences in domestic fowl breeds were determined using machine learning algorithms based on morphometric measurements obtained from the tarsometatarsus bone. The developed model revealed breed differences in the tarsometatarsus of two different domestic fowl breeds. In addition to breed differences, the current model revealed the strongest distinguishing features among morphometric measurements. It was shown that breed differences can be determined quickly and accurately using a minimum number of measurements taken from the tarsometatarsus. This developed model combines morphometric data obtained from bones with the latest advancements in computing, offering an innovative method for scaling, accelerating, or improving applications in this field.

## 1. Introduction

In recent years, learning model methods such as computer vision and deep learning have begun to come to the forefront in the measurement and classification of bones in avians [1]. Among avian species, domestic fowl (*Gallus gallus domesticus* L. 1758) is widely raised. Domestic fowl is the earliest domesticated avian and is found on every continent in the world, except for Antarctica. It has become a dominant part of our diet in recent years [2,3,4]. It is accepted that the Celts were responsible for the spread of the domestic fowl, which was domesticated in Asia, while the Greeks and Romans domesticated it in larger numbers [4,5,6].

In recent years, gender and breed differences have begun to be examined in avian species, as in mammals, using measurements obtained from bones [7,8]. Different measurement methods are used for this purpose. The most frequently used of these methods are morphometric measurements obtained directly from bones or from radiological images, and geometric morphometric methods performed on bones or three-dimensional models [7,9,10,11,12,13,14]. In order to determine gender, age, and breed differences in avians, the tarsometatarsus, one of the hindlimb bones, is often examined [15,16,17,18,19]. In avians, the tarsometatarsus extends from the base of the toes to the intertarsal joint. It is formed by the elongation and lateral fusion of three cylindrical periosteal bones [16]. The tarsometatarsus bone, located within the skeleton pedis, is shaped by the fusion of metatarsal bones II–IV and the distal row of the tarsal bones [20,21]. The assembly of the constituent elements of the tarsometatarsus bone begins in ovo, and ossification occurs in the early postnatal period. The tarsometatarsus expands rapidly after hatching, doubling in length within the first month, with longitudinal growth completed between 3 and 6 months. Even after reaching the maximum length, its diameter continues to increase for 1–2 months [15,22,23,24].

Various studies have examined breed and gender differences in domestic fowl using morphometric measurements of the tarsometatarsus [15]. Furthermore, differences have been identified by examining tarsometatarsus from different historical periods [3,18]. Although morphometric measurements of the tarsometatarsus in domestic fowl are limited, measurements have been made in various avian species. Morphometric measurements were carried out on the tarsometatarsus of pigeons (*Columba livia*) [7,25], terrestrial birds [19], ostriches (*Struthio camelus*) [26], Indian eagle owl (*Bubo bengalensis*) [27], and waterfowl (*Anseriformes*) [14].

Long bones such as the tarsometatarsus in avians were compared using different measurement methods (digital caliper, laser scanner, and µCT). Differences in 2D/3D measurements of long bones were evaluated, and the consistency between measurements was tested. Thus, important background data was provided for the calibration and validation of artificial intelligence-supported systems. This method used U-Net and Mask R-CNN architectures to segment bone pixel clusters in images, automatically identify bone type, and calculate the longest linear dimension to create a large-scale morphometric dataset [28,29]. A deep neural network-based method called “Skelevision” has been developed to perform automated morphometric measurements from photographs of avian bones found in museum collections [1,29]. In addition to morphometric measurements, artificial intelligence evaluation is also performed on data obtained from geometric morphometry, a method frequently used in studies examining the relationship between avian morphology, ecological niches, and paleoecology. A comprehensive study of nine extinct waterfowl species obtained a large geometric morphometric dataset from skull and long bones. The dietary ecologies of these species have been estimated with high accuracy using methods such as random forest (RF) and linear discriminant analysis [30]. This approach demonstrates how powerful predictions can be made when morphometry and geometric morphometry data are analyzed using machine learning. In another study combining artificial intelligence and geometric morphometry, machine learning was shown to be successful in classifying cut marks, bone surface features, and other morphological structures. Shape data obtained from cross-sections using geometric morphometry was combined with random forest, support vector machines (SVMs), and artificial neural networks to enable highly accurate classification of the type and formation process of cut marks [31]. This shows that a similar approach could be applied to determine the species, breed, or gender of avian bones.

While numerous studies in the literature highlight the ecological and evolutionary significance of tarsometatarsus morphology, the number of studies integrating linear morphometric data of this bone with machine learning for the differentiation of hybrid breeds is quite limited. Many current studies focus on interspecies ecomorphological comparisons or automated measurement and macro-ecological analysis of datasets with broad taxonomic coverage. From this perspective, the present study focuses on differentiating two different hybrid chicken breeds through a single long bone (tarsometatarsus). This differentiation is carried out by combining precise morphometric measurements obtained with a digital caliper and machine learning algorithms.

This study aims to quantitatively demonstrate breed discrimination and morphometric differences. In this respect, it fills a gap in the existing geometric morphometry–ecology-focused literature by complementing veterinary production practice and the morphological differentiation of hybrid breeds. Furthermore, instead of using many morphometric parameters as in other methods for distinguishing bones, the aim is to identify the strongest discriminant measurements with the help of machine learning.

## 2. Material and Methods

### 2.1. Animal Collection

In this study, tarsometatarsus from two different meat-type hybrid breeders were used. The study material was carefully obtained from two different poultry farms that had similar housing, nutrition, and growth conditions from hatching to adulthood. Particular attention was paid to ensuring that the farms from which the materials were obtained had similar characteristics. Adult (over 45 weeks), healthy, female right and left tarsometatarsus bones of Ross and Cobb hybrid breeds (81 and 83, respectively) were used. A total of 328 tarsometatarsus bones belonging to two different domestic fowl breeds were used in the study. For the reliability of the results, care was taken to ensure that the collected tarsometatarsus bones belonged to fully grown animals. Similarly, care was taken to ensure that the animals whose bones were used had similar housing, nutrition, and growth conditions from hatching to adulthood. The study was approved by the Ethics Committee of Selçuk University Faculty of Veterinary Medicine (approval number: 2025/152). The bones were subjected to a maceration process, which removed all soft tissue, at the Department of Anatomy.

### 2.2. Morphometric Measurement of Bones

**Figure 1:**
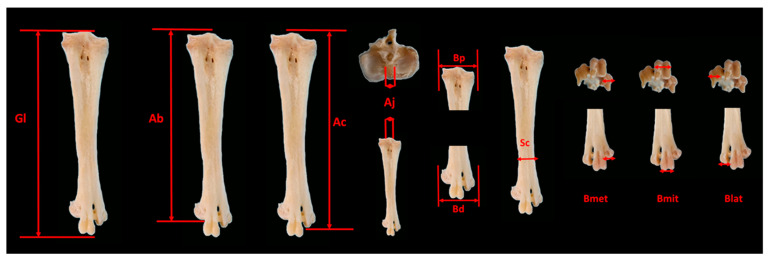
Morphometric measurements performed on the tarsometatarsus. (Gl) Greatest length. (Ab) Extension of the *trochlea metatarsi II.* (Ac) Extension of the *trochlea metatarsi IV.* (Aj) Latero–medial *hypotarsus width.* (Bp) Breadth proximal. (Bd) Breadth distal. (Sc) Smallest diameter of the corpus. (Bmet) Breadth medial trochlea. (Bmit) Breadth of middle trochlea. (Blat) Breadth of lateral trochlea.

After removal of the soft tissue fragments, measurements were made on the tarsometatarsus using a 0.01 mm accuracy digital caliper. Ten different morphometric measurements, as shown in Figure 1, were performed in line with the reference points specified in previous studies [15,18,19,25,26,27,32,33]. Morphometric measurements were meticulously performed by the same researcher to eliminate inter-observer errors. In total, 10 different morphometric measurements were performed on 328 tarsometatarsus bones, and 3280 data points were obtained.

### 2.3. Application of Machine Learning Algorithms

Morphometric measurements obtained from the tarsometatarsus of Ross and Cobb meat-type hybrid breeders were analyzed. Machine learning was used considering the dataset’s density and suitability.

#### 2.3.1. Dataset and Morphometric Variables

In this study, the raw dataset consisted of morphometric measurements obtained from 328 tarsometatarsus bones of Ross (*n* = 162) and Cobb (*n* = 166) domestic fowl. For each sample, a series of linear morphometric measurements were taken, including total and partial lengths and widths (Gl, Ab, Ac, Aj, Bp, Bd, Sc, Bmet, Bmit, and Blat) along the tarsometatarsus. Additionally, a categorical variable defining the side of the bone (direction information, coded as right and left) was also available. All the data in the dataset was used, and the necessary cleaning process was performed in the next step.

The binary response variable constitutes the population label (Breeds: Ross vs. Cobb). An artificial identity column was created for indexing, but it was excluded from all models. After cleaning, necessary assessments were conducted, and gaps were identified to ensure that no missing measurement values remained (full case analysis, *n* = 326). The dataset used consisted of measurements obtained from 161 Ross and 165 Cobb tarsometatarsus bones.

All data processing and analyses were performed in the R programming language (Version 4.5.1). Various packages were used (packages: readxl [1.4.5], dplyr [1.1.4], janitor [2.2.1], caret [7.0-1], randomForest [4.7-1.2], ggplot2 [4.0.1], pROC [1.19.0.1], corrplot [0.95], rrcov [1.7-7], car [3.1-3], and tibble [3.3.0]).

#### 2.3.2. Pre-Processing and Data Partitioning

Column names were standardized to lowercase and snake_case. Variables with lateral (direction) levels (“right”, “left”) were converted into factors.

All other character variables (if any) were converted into factors, while morphometric measurements were treated numerically.

The dataset was partitioned into training (70%; *n* = 229) and test (30%; *n* = 97) subsets using the caret package’s createDataPartition command, stratified by class label (breeds). The Ross population was designated as the reference (positive) class in the binary classification.

#### 2.3.3. Machine Learning Models and Cross-Validation

We evaluated three classical supervised learning algorithms for binary discrimination between Ross and Cobb populations:

1. **Random forest (RF)**
  ○ Implemented using caret::train with method = “rf”.
  ○ Number of trees: ntree = 500.
  ○ The mtry hyperparameter (number of variables randomly sampled at each split) was tuned over a small grid around the theoretical √*p* rule: mtry ∈ {2, 3, 4} (with *p* = 12 predictors).
  ○ No pre-processing was applied to predictors.
2. **Support vector machine with radial kernel (SVM-RBF)**
  ○ Implemented using method = “svmRadial”.
  ○ Predictors were centered and scaled prior to model fitting.
  ○ The cost parameter C ∈ {0.25, 0.5, 1.0} was tuned, while the kernel width (sigma) was estimated by *caret* and held constant across resamples.
3. **Generalized linear model (GLM, logistic regression)**
  ○ Implemented using method = “glm” with binomial link.
  ○ No explicit pre-processing was used.

For all three models, hyperparameters were tuned using 10-fold cross-validation with class probabilities enabled and ROC as the primary performance metric (trainControl (method = ‘cv’, number = 10, classProbs = TRUE, summaryFunction = twoClassSummary)).

#### 2.3.4. Model Evaluation on the Test Set

For each fitted model (RF, SVM, GLM), we generated class predictions and class probabilities on the independent test set. Performance was quantified via:

- Overall accuracy and Cohen’s kappa;
- Sensitivity (recall for Ross);
- Specificity (correct classification of Cobb);
- Precision (positive predictive value);
- F1-score (harmonic mean of precision and recall);
- ROC curves and AUC values (using pROC);
- Confusion matrices were obtained using caret::confusionMatrix.

#### 2.3.5. Variable Importance and Feature Selection

To quantify the relative contributions of morphometric variables, variable importance was calculated using random forest-based caret::varImp.

Importance scores were obtained per class and averaged to obtain an overall “total” score.

Additionally, recursive feature elimination (RFE) was applied with RF as the base learner (rfeControl (functions = rfFuncs, method = ‘cv’, number = 10)). Subset sizes ranging from 2 to the number of all predictors were evaluated, and the subset yielding the highest cross-validation accuracy was selected.

#### 2.3.6. Correlation Analysis, Dimensionality Reduction, and Multicollinearity

To investigate the interdependencies between morphometric measurements, a Pearson correlation matrix was calculated for all numerical variables (excluding ID). A heatmap was generated using the corrplot command.

To address multicollinearity, strong correlation predictors were identified in the training set using caret::findCorrelation with a cut-off value of |r| > 0.90. Variables flagged by this procedure were removed, and the RF and GLM models were re-fitted and rebuilt using this reduced feature set.

We further explored the structure of the morphometric space via:

- Classical Principal Component Analysis (PCA) on standardized numeric variables (prcomp, center = TRUE, scale. = TRUE);
- Robust PCA using PcaHubert (package *rrcov [1.7-7]*), which down-weights potential outliers and yields robust scores and loadings.

Scatter plots of PC1 vs. PC2 for both PCA and robust PCA were drawn, coloring points by population (Ross vs. Cobb) and adding 95% confidence ellipses.

#### 2.3.7. Reduced and Feature-Selected Models

Two additional sets of models were considered:

1. Reduced models (RF_Reduced, GLM_Reduced)
  ○ Built after removing features with |r| > 0.90 identified in the correlation analysis.
2. Feature-selected models (RF_FS, SVM_FS, GLM_FS)
  ○ Built using only the RFE-selected subset of predictors.

All models were evaluated in the relevant test subsets, and performance metrics are summarized in a single comparison table.

**Figure 2:**
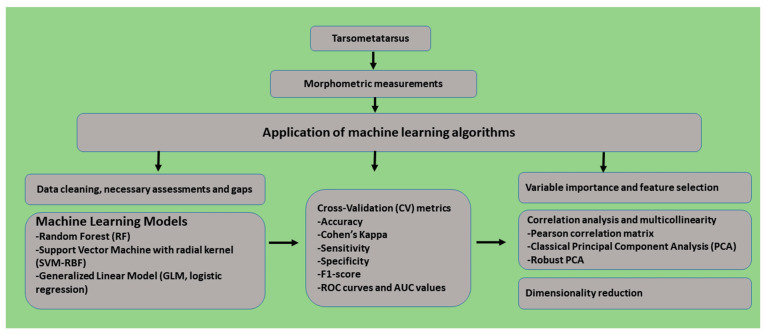
The workflow for a model created using machine learning.

Finally, to further quantify the multicollinearity among predictors, variance inflation factors (VIFs) were calculated for the full logistic regression model (GLM). The workflow of the model developed based on tarsometatarsus measurements and machine learning for the identification of domestic fowl breeds is presented in Figure 2.

## 3. Results

### 3.1. Model Performance on the Test Set

All three baseline models—RF_full, SVM_full, and GLM_full—achieved perfect discrimination between the Ross and Cobb populations on the independent test set (*n* = 97; 48 Ross, 49 Cobb):

- Accuracy = 1.000;
- Kappa = 1.000;
- Sensitivity = 1.000;
- Specificity = 1.000;
- F1-score = 1.000 for Ross as the positive class.

The 95% confidence interval for accuracy was [0.963, 1.000], indicating statistically robust classification despite the relatively small test set.

**Figure 3:**
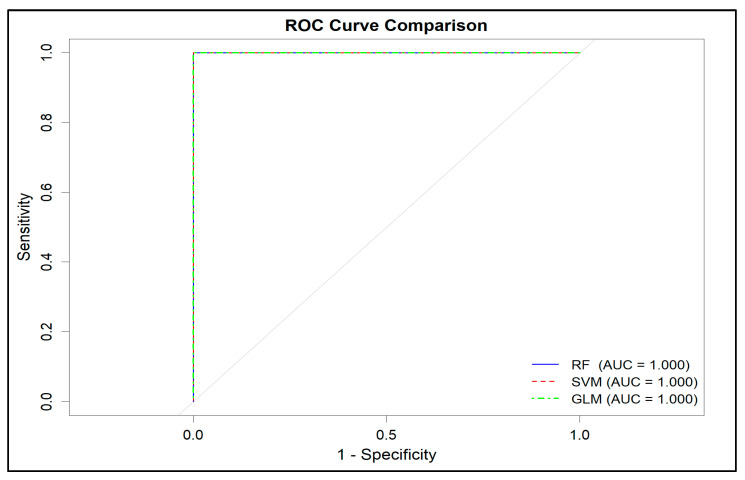
ROC curves and corresponding AUC values for the RF, SVM, and GLM classifiers on the test dataset. The ROC curves are **fully overlapping**, as all three models yielded identical AUC values (AUC = 1.000), indicating equivalent classification performance on the test set.

The cross-validated ROC performance on the training set was also excellent. While the cross-validated ROC for RF and SVM was 1.000, the logistic regression model achieved an ROC ≈ 0.984. The ROC curves calculated on the test set showed that the AUC values for all three classifiers were essentially equal to 1.0, confirming that the two populations were almost perfectly separated in the morphometric space (Figure 3).

It should be noted that the GLM issued numerical warnings during fitting (“the algorithm did not converge; numerical probabilities of 0 or 1 emerged”), and this situation is consistent with the expected perfect separation given the very strong classification results.

### 3.2. Variable İmportance and Discriminative Morphometrics

**Figure 4:**
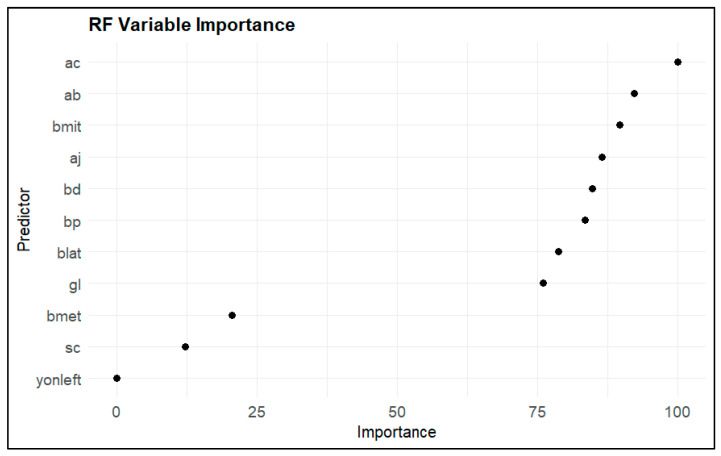
Random forest variable importance analysis.

Random forest variable importance (average across classes) revealed a clear hierarchy of discriminative features (Figure 4). The most informative predictive factors were Ac (extension of the *trochlea metatarsi IV*), Ab (extension of the *trochlea metatarsi II*), and Bmit (breadth of the middle trochlea), followed by Aj (latero–medial *hypotarsus width*), Bd (breadth distal), and Bp (breadth proximal). Blat (breadth of the lateral trochlea) and Gl (greatest length) contributed moderately, while Bmet (breadth of the medial trochlea) and Sc (smallest diameter of the corpus) showed a relatively limited effect.

In contrast, the laterality variable (left and right) was of negligible importance (≈0), indicating that bilateral asymmetry did not contribute significantly to the distinction between the Ross and Cobb populations; classification was driven by morphometric differences related to size and shape rather than laterality.

**Figure 5:**
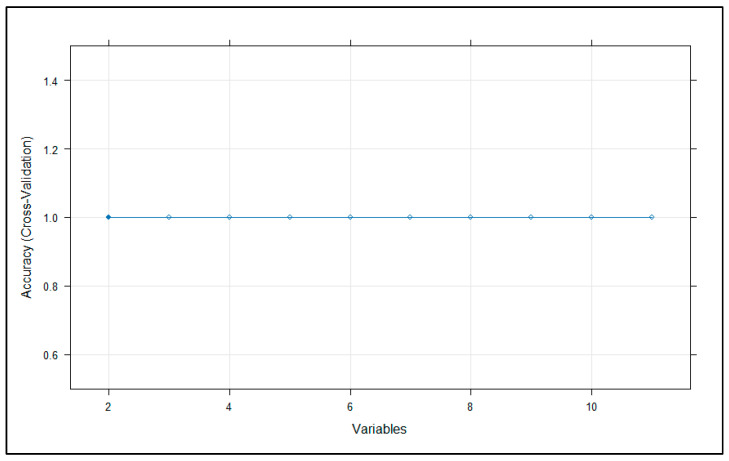
The recursive feature elimination (RFE) method.

Consistent with this, the recursive feature elimination (RFE) method showed that a minimal subset of only two variables (ac and bmit) was sufficient to achieve 100% cross-validated accuracy (Figure 5). This finding demonstrates that a very small number of tarsometatarsal measurements capture nearly all of the discriminative signals between the two populations.

### 3.3. Correlation Structure and Multicollinearity

**Figure 6:**
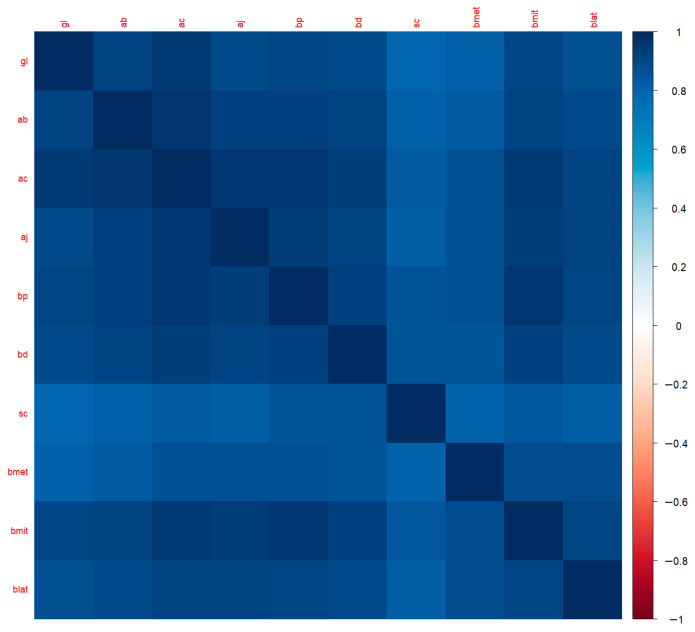
Pearson correlation heat map of measurements.

The Pearson correlation matrix of morphometric variables revealed very strong positive relationships between most length and width measurements, with many pairs exceeding ∣*r*∣ > 0.95 (Figure 6). There was a high correlation particularly between Gl, Ab, Ac, Bp, Bmit, and Blat, and the correlation coefficients were generally found to range between 0.90 and 0.96. This indicates that these measurements capture the common dimensional component of the tarsometatarsus rather than largely independent shape information.

To reduce redundancies, the findCorrelation function was applied to the training set using a threshold value of ∣*r*∣ > 0.90. This procedure identified Ac, Bp, and Bmit as the most redundant predictors, which were subsequently removed to form the reduced feature set.

Consistent with the correlation structure, the variance inflation factor (VIF) diagnoses obtained from the full logistic regression model confirmed severe multicollinearity with significant inflation for several predictors (e.g., Bp, Ac, Blat, Bmit, Ab, and Sc), while the remaining variables showed relatively lower inflation. Collectively, these results provide strong statistical justification for excluding highly correlated predictors in alternative model specifications to improve interpretability and coefficient stability.

### 3.4. Principal Component Analysis and Robust PCA

Classical PCA performed on standardized morphometric variables revealed a structure dominated by dimension. The first principal component (PC1) alone explained 90.30% of the total variance, while PC2 accounted for 2.64%, with the first two components together explaining 92.96% of the variance. The first three components together explained 94.99% of the variance, while the first five components accounted for 97.14% of the cumulative variance.

**Figure 7:**
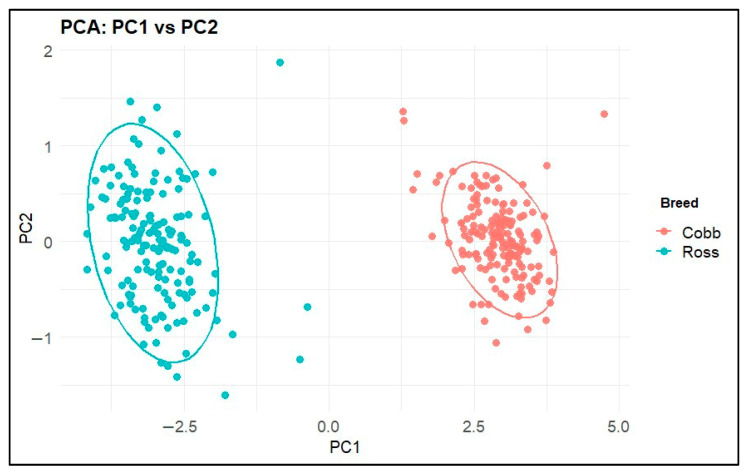
Results of classical PCA analysis performed on morphometric variables.

PC1 showed strong and largely similar loadings across the main length and width measurements (Gl, Ab, Ac, Bp, Bd, Sc, Bmet, Bmit, and Blat), consistent with the dominant general size axis of the tarsometatarsus (Figure 7). In contrast, PC2 contributed to only a small portion of the total variance (2.64%) and therefore represented subtle shape variation relative to the dominant size signal; its biological interpretation should be considered secondary to that of PC1.

Robust PCA performed using the Hubert predictor (PcaHubert) yielded results fully consistent with the classical PCA and confirmed that the dominant source of variation was a strong common dimension component. According to the robust component eigenvalues, PC1 accounted for approximately 94.2% of the total variance captured by the robust model, while PC2 explained only ~2.35% (each of the remaining components contributed <2%; the cumulative variance of the first two components was ≈96.5%).

**Figure 8:**
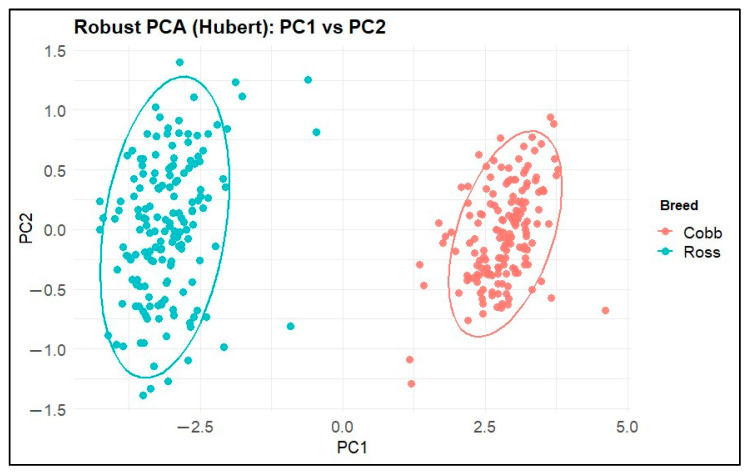
Results of robust PCA (PcaHubert) analysis performed on morphometric variables.

In the robust PC score space (PC1–PC2), the Ross and Cobb samples formed two clearly separated clusters, primarily along PC1, with minimal overlap between the 95% ellipses (Figure 8). This clear separation in the reduced morphometric space supports the nearly perfect classification performance observed in the controlled models and indicates that group differences are dominated by a strong underlying morphometric (size/shape) signal rather than outliers or leverage points.

### 3.5. Performance of Feature-Selected and Reduced Models

Model performance was equally high across all features, indicating that the classification is robust in terms of both feature selection and correlation-based reduction. All three full-featured models (RF_full, SVM_full, and GLM_full) achieved excellent test set performance (accuracy = 1.000, kappa = 1.000, sensitivity = 1.000, specificity = 1.000, F1 = 1.000), and RF_full was selected as the best model according to the applied accuracy criterion.

Consistent with this, the correlation-reduced models (RF_Reduced and GLM_Reduced, after removing highly correlated predictors such as Ac, Bp, and Bmit) also achieved excellent classification on the test set (all metrics = 1.000). The RFE-based reduced models similarly remained robust: GLM_FS achieved excellent performance, while RF_FS and SVM_FS showed a marginal decline (accuracy = 0.99, kappa = 0.979, specificity = 0.98, F1 = 0.99) yet maintained a sensitivity of 1.000. Overall, these results indicate that the discriminative signal between Ross and Cobb is quite distinct and can be captured under the current evaluation scheme using either the full measurement set or a significantly reduced subset of predictors.

In terms of parsimony and interpretability, the results can be summarized as follows:

Multi-modeling strategies, including tree-based (RF), kernel-based (SVM), and linear (GLM) classifiers, demonstrate an exceptionally strong discriminative signal, as they perfectly separate the two populations under the current evaluation scheme when using the full set of predictors.

Only a very small subset of measurements is required to maintain this performance. In accordance with the variable importance ranking, Ac was the most informative single predictor, with Bmit and Ab also making a strong contribution. Furthermore, RFE identified a minimum two-variable subset (Ac and Bmit) capable of achieving 100% cross-validation accuracy and highlighted that most of the discriminative information was concentrated in a few key morphometric dimensions.

Reducing predictor redundancy enhances interpretability without compromising accuracy. Given the evident multicollinearity among raw measurements (supported by both the correlation structure and VIF diagnoses), removing highly correlated predictors provides a statistically justified path to more stable and interpretable inference models (e.g., logistic regression) while maintaining excellent predictive performance. Although tree-based methods are generally more tolerant of multicollinearity, this is still recommended.

## 4. Discussion

In avians, measurements obtained from bones are used to determine species, breed, and gender. These findings are very important for distinguishing breeds [7,8,11,14]. The present study used an innovative method to determine breed differences using the tarsometatarsus. While differences between morphometric measurements obtained from bones using traditional methods were revealed, the present study identified specific differentiating measurements using machine learning algorithms. Morphometric measurements obtained from bones were examined to determine which of them are most important for achieving clear differentiation. The developed model shows that it is possible to determine which breed of domestic fowl a particular tarsometatarsus belongs to, and it can be applied to different bones after further development. Thus, it is thought that breed differences in domestic fowl can be determined more quickly and reliably.

In domestic fowl, the tarsometatarsus is one of the primary bones for determining gender and breed. The presence of a spur on this bone is an important reference point for gender differentiation [3,15,16,17,18]. In the present study, the tarsometatarsus bone was specifically used, taking into account these findings in the literature. As is well known, the spur allows for gender differentiation in domestic fowl; however, since the present study focuses on breed differentiation, the spur on the tarsometatarsus was not included in the scope of the study. Furthermore, the fact that the spur is damaged or missing in some of the bones found in archaeological excavations is another reason for its exclusion. Various studies are being conducted to determine the breed of domestic fowl bones obtained from archaeological excavations. These studies utilize measurements obtained from long bones. As stated in the present study, the tarsometatarsus is the most decisive bone when determining breed and gender from long bones [3,18]. A previous study distinguished ancient breeds by combining the use of frequency histograms of the lengths of the tarsometatarsus and other long bones with observations of medullary bone development and admixture analysis. GL measurement of the tarsometatarsus and other long bones was used to differentiate medullary bone development [18]. In the relevant study, medullary bone development, along with morphometric measurements, played a significant role in identifying differences. Measurements obtained from many bones were used for breed differentiation. This innovative model demonstrated that breeds can be differentiated more quickly and effectively by using only the strongest differentiating morphometric measurements obtained from the tarsometatarsus. Similar to the present study, attempts have been made to identify breed differences in the bones of other avian species using morphometric measurements. Based on measurements obtained from different bones, the tarsometatarsus was found to be one of the most significant bones in identifying differences [7,14,19,25,26,27].

Geometric morphometry, distinct from linear measurements, reveals variations in bone shape and form. This method uses landmarks to identify anatomical variations in bone structures [9,10,11,12,13,14]. In the model, which was trained and tested with different machine learning algorithms, the most important morphometric measurements for breed differentiation were determined. Thus, it was shown that, when trying to differentiate between two different breeds, data obtained from the strongest distinguishing morphometric measurements would suffice, instead of measuring all morphometric parameters that can be obtained from the bone. While geometric morphometry methods identify anatomical structures with the highest variation, this study identified the strongest measurements revealing differences. The most important differences in the tarsometatarsus of different breeds were identified. Thus, the developed model shows that it is possible to determine which breed of domestic fowl any given tarsometatarsus bone belongs to. Beyond revealing breed differences in any bone using this model, by expanding the dataset, it will be possible to directly identify which breed a bone belongs to.

Differences between species of waterfowl (Anseriformes) have been determined using geometric morphometry. It is stated that the data obtained from geometric morphometry analysis of the tarsometatarsus is useful for reliably evaluating the locomotory habits of fossil waterfowl [14]. A relevant study observed that the first three variation values of the PCA for shape variation were lower than the PCA value in the current study. The method in the current study achieved a clearer distinction between breeds than PCA and demonstrated perfect classifier performance. In terrestrial birds, PC1 and PC2 explain the variation in three different variables, while the present study shows strong equal loadings for all relevant variables [19]. The results indicate that machine learning algorithms yield more accurate and precise outcomes in detecting breed differences. It has been demonstrated that breed-level morphological differences in bones can be identified effectively and efficiently.

A deep neural network was developed based on data of the long bones of museum skeletal specimens obtained using different measurement methods (digital caliper, laser scanner, and µCT). The analysis focused on 11 functional traits encompassing the length of the tibiotarsus, humerus, metatarsus, ulna, radius, keel, carpometacarpus, second digit first phalanx (hereafter, digit P1), and femur. Consistency between measurements was tested, providing important background data for the calibration and validation of AI-powered systems [1,28,29]. Machine learning was used in the present study, considering data density and suitability. RF, SVM-RBF, and GLM logistic regression algorithms were used to determine the difference between two different breeds. In all algorithms used, the differences between the two breeds were clearly revealed. It was demonstrated that, by expanding the model’s database with measurements obtained from the tarsometatarsus bones of domestic fowl breeds, the breed to which a bone belongs can be quickly and accurately determined using only two measurements (Ac and Bmit) from the tarsometatarsus. In domestic fowl breed classification, it has become possible to reliably identify breeds using machine learning algorithms based on the tarsometatarsus bone. This developed model combines morphometric data obtained from fields such as veterinary anatomy and taxonomy with the latest advances in computer vision, offering an innovative method for scaling, accelerating, or improving applications in science. Developing such methods requires careful evaluation and collaboration to formulate the problem and determine which of the various artificial intelligence models is suitable. In this model example, this collaboration guided how the data was collected, annotated, segmented, and measured to achieve the final results. The model was tested not with a single machine learning algorithm but with different algorithms (RF, SVM-RBF, and GLM logistic regression), all yielding highly reliable results. Furthermore, considering that this developed model can also be used in other avian species and mammals, it is evident that breeds can be differentiated using morphometric measurements and geometric morphometry data obtained from bones using different methods (digital caliper, 2D images, and 3D models).

A comprehensive study of nine extinct waterfowl species has generated a large geometric morphometric dataset of the skull, femur, tibiotarsus, and tarsometatarsus. The dairy ecologies of these species were estimated with high accuracy using RF and linear discriminant analysis (LDA) methods. For the tarsometatarsus, PC1 described its relative elongation, while PC2 largely defined the width of the trochlea, the location of the trochlea II, and the length of the crista hypotarsi [30]. In the present study, PCA performed on standardized morphometric variables revealed a structure dominated by dimension. In machine learning algorithm evaluations of data obtained from two different measurement methods, the prominence of closely related and similar anatomical regions of the tarsometatarsus indicates that the relevant algorithms yielded highly accurate results. Another study combining artificial intelligence and geometric morphometry demonstrated that machine learning could classify cut marks, bone surface features, and other morphological structures with high accuracy. The study indicated the period to which the cut marks belonged and the types of tools used to produce them [31]. Similar to the current study, this classification was performed with high accuracy using RF and SVM. In the current study, where breed differences were revealed from measurements of the tarsometatarsus bone using machine learning algorithms, it was shown that these algorithms can reveal not only shape variations but also differences in morphometric measurements with high accuracy.

## 5. Conclusions

Breeds were discriminated and the strongest discriminants were identified using machine learning and the tarsometatarsus of different domestic fowls. Machine learning models provide highly reliable and explanatory results, particularly when considering multiple and correlated variables together. This study demonstrates that using machine learning algorithms to distinguish breed and orientation via morphometric measurements enables highly accurate and reliable classification. Among the morphometric measurements, Ac and Bmit were found to be the strongest distinguishing features.

It is thought that enriching the dataset of the obtained model with data from different avian breeds will enable breed determination in the future using only the tarsometatarsus and will contribute to studies in this field. Furthermore, considering that this model can also reveal differences with high accuracy by using morphometric measurements obtained from mammalian bones via digital calipers or 3D models, it is evident that the model has a wide application range.

## Data Availability

The R scripts used in this study are publicly available in the project repository (https://github.com/mzeybek583/tarsometatarsus-ross-cobb-ml.git, accessed on 2 February 2026). The raw datasets supporting the findings of this article are available from the authors upon reasonable request.
